# Submandibular Approach for Tracheal Intubation – A Case Report

**Published:** 2009-02

**Authors:** G Uma, P N Viswanathan, P S Nagaraja

**Affiliations:** 1 Lecturer, Department of Anaesthesiology, JSS Medical College & Hospital, Mysore – 570004; 2 Professor & HOD, Department of Anaesthesiology, JSS Medical College & Hospital, Mysore – 570004; 3 P.G. Student, Department of Anaesthesiology, JSS Medical College & Hospital, Mysore – 570004

**Keywords:** Submandibular intubation, Maxillofacial surgeries, Panfacial fractures, Le Fort's fracture

## Abstract

**Summary:**

Intubating a patient with panfacial fractures is always a challenge to the anaesthesiologist. In a 40-yr-old male patient with left Le Fort's III fracture with nasal bone and symphysis menti fracture, we successfully carried out oral endotracheal intubation which was then modified to submandibular approach to provide adequate surgical field. Initially oral endotracheal intubation was performed, then an incision was made in the submandibular region through which the endotracheal tube was brought out and maintained as submandibular approach throughout the surgery.

## Introduction

Maxillofacial surgical patients present a specific challenge to the anaesthesiologist. The standard oral route for tracheal intubation can be unsuitable for some maxillofacial surgeries because it can interrupt the surgical field and can interfere with teeth occlusion frequently needed for the adjustment and fixation of maxillary fractures.^1^ The nasal route can be used, however it can be impossible as a result of a deformity or fractures in nasal bone and it may interrupt surgical accessibility. Also naso-tracheal intubation is contraindicated in fractures of cribriform plate of ethmoid, which frequently accompany LeFort II or III maxillay fractures because of potential complication of infection and the possibility of cranial intubation. The standard solution in these situations is to perform an elective short term tracheostomy before the surgery. Since there is high risk of morbidity associated with tracheostomy an alternative method is to introduce a tracheal tube via a submental incision which was first introduced by Sir Hernandez Altemir in 1986.^2^,^3^

The submandibular intubation which we have performed is a modification of submental intubation and is a better alternative method for short term tracheostomy.^4^

## Case report

A 40-yr-old ASA grade III male patient had met with a road traffic accident and sustained panfacial fractures. He was evaluated by the maxillofacial surgeons and posted for open reduction and internal fixation of the fractures.

His pre-anaesthetic evaluation revealed history of loss of consciousness for a brief period of less than five minutes with no history of vomiting or seizures. He had no other significant medicalor surgical illnesses. On examination he was a moderately built and nourished middle aged male patient with stable vitals and normal cardiovascular and respiratory systems. He was conscious and oriented to time, place and person. His airway assessment revealed a restricted mouth opening of one and half fingers (due to pain). Mallampatti scoring couldn't be assessed because of restricted mouth opening. The temporomandibular joint mobility was restricted. Both the nares of the patient were patent with bilateral equal free flow of air on forced expiration. His laboratory investigations revealed a normal hemogram and coagulation profile. X-rays were suggestive of maxillary, nasal and symphysis fractures. CT also suggested the same.

We planned for submandibular approach for endo-tracheal intubation after discussing with oromaxillo-facial surgeon. The procedure was explained to the patient and his relatives in their own native language and written informed consent was obtained. Standby for possible tracheostomy in the event of any emergency was available.

In the operating room 18 gauge IV cannula was secured in the left arm. Monitoring included ECG, pulse oximeter and non-invasive blood pressure.

**Fig 1:**
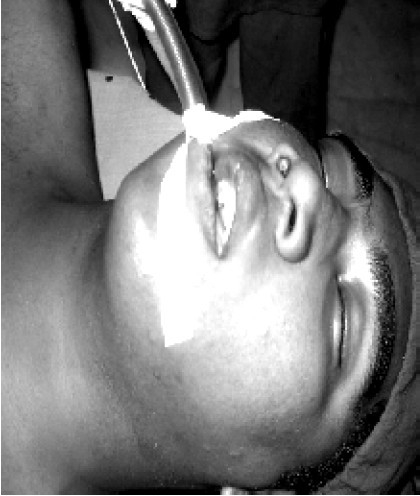
After oral intubation

Glycopyrrolate 0.2 mg, ondansetron 4 mg, pethidine 60 mg were administered intravenously as premedication. Patient was preoxygenated with 100% oxygen for 3 minutes and anaesthesia was induced with thiopentone sodium 300mg intravenously. Suxamethonium 100mg intravenously was administered as the muscle relaxant for intubation and intubated with 8.0 mm ID flexometallic cuffed endotracheal tube, connected to Bain's circuit. The tube was secured in place after confirming bilateral equal air entry (Fig 1). Throat was adequately packed. Anaesthesia was maintained with 60% nitrous oxide, 40% oxygen and halothane 0.5 – 1%. Controlled ventilation was facilitated by vecuronium bromide.

Subsequently the surgeon created a track from the oralcavity extending to the submandibular region. The side of the submandibular area used was dictated by the presence of mandibular fracture, however in our case, right side was preferred as the patient had left sided fractures.

**Fig 2:**
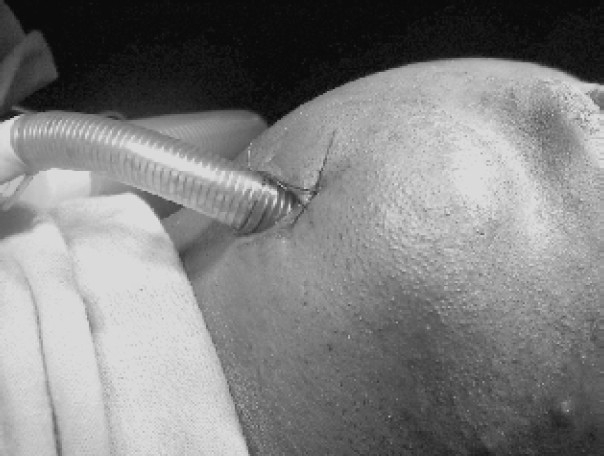
Position of the endotracheal tube after submandibular intubation

A 1.5cm transverse skin incision was made in the submandibular area, about 1 inch below and half an inch anterior to the angle of the mandible. This distance from the lower border of the mandible was to avoid an injury to the mandibular branch of the facial nerve. Using a curved artery forceps, blunt dissection was carried out through the skin incision in the upward direction towards the oral cavity. The subcutaneous fat, platysma, investing layer of the deep cervical fascia and the mylohyoid muscle were dissected until the tip of the artery forceps tented the mucous membrane of the oral cavity. The end of the pilot balloon of the endotracheal tube cuff was grasped with the tip of theartery forceps and then pulled through the dissected track to come out through the submandibular incision. The tracheal tube then disconnected from Bain's circuit and its tube connecter was removed. Under direct vision using the laryngoscope and with the tube supported in the oro-pharynx by the tip of the assistant's index finger, the end of the tracheal tube was grasped by the tip of the artery forceps and pulled out through the submandibular incision, in the same way as the pilot balloon of the endotracheal tube cuff. The tube was supported in the oro-pharynx throughout to prevent accidental extubation or inward pushing of the tube. Any blood present was suctioned and throat pack was changed to a fresh one. After checking the tube position, a mark was made on the tube at the skin exit site. A silk stay suture was made to fix the tube to the skin in the submandibular region (Fig 2). The tube was further secured by adhesive tape applied circumferentially. Anaesthesia was maintained with 60% nitrous oxide, 40% oxygen and halothane 0.5 – 1%. Controlled ventilation was facilitated by vecuronium bromide.

At the end of the surgery, the stay sutures and adhesive plaster fixing the tube were removed and tracheal tube was pulled back to the oral cavity, followed by the pilot tube. The submandibular incision was sutured and bandaged. In our patient, the oral endotracheal tube was retained post operatively and subsequently extubated.

## Discussion

The submental route for tracheal intubation was first introduced by Sir Hernandez Altemir in 1986^2^. This technique provides a secure airway whilst at the same time allowing an unobstructed surgical field for adequate reduction and fixation of midface and panfacial fractures.^5^,^6^ Submental tracheal intubation also avoids the potential complications associated with nasal intubation and tracheostomy.^3^

Stoll described a similar technique to submental intubation but where the incision is placed further posteriorly in the submandibular region and Prochno reported 14 patients who underwent submanbular transmylohyoid intubation.^7^ Submandibular intubation is a modification of submental intubation. It was found to be an easy and convenient technique which avoids the potential complications of submental approach like, damage of sublingual and submaxillary ducts, sublingual gland and lingual nerve. Pulling the end of the tube through the deep cervical fascia in the submandibular area may probably be easier than in the tight submental area^1^.

**Fig 3:**
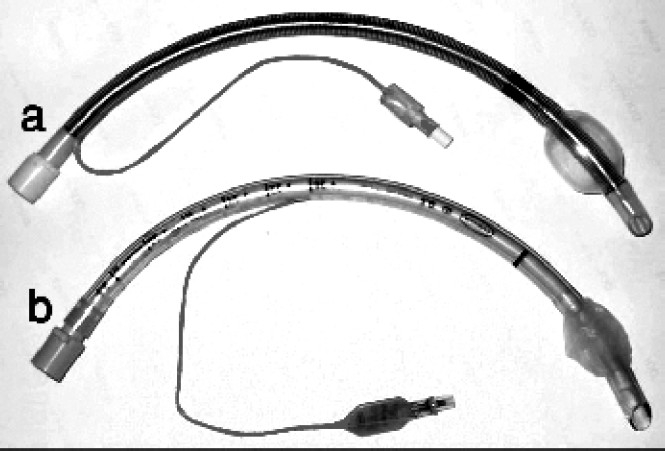
(a) Armoured and (b) regular portex endotracheal tubes

It has been found that some of the armoured tubes (Fig 3) have connectors not designed to be removed. The end of such tubes has to be cut off and when reattached forms a loose connection. More recently armoured tubes having a connector that is specifically designed for detachment and re-attachment are available making it ideal for submandibular tracheal intubation.

Partial dislodgement of the submandibular tube, wherein the tube being pushed down the right main bronchus, bleeding and skin infection are some of the complications that have been reported.

The position of the tube being pushed down the right main bronchus is probably due to the tube not being secured properly to the skin. Partial extubation of the tube was a common documented complication. Securing the tube by both circum ferential adhesive tape and skin sutures and by doing the submandibular positioning of the tube under direct laryngoscopic vision prevented the above complications.

Studies report submandibular tubes to be converted to oral tubes at the end of surgery and most of them extubated at the theatre. Delayed oral extubation in the intensive care unit also is reported because of associated facial injury. In our patient also because of the facial swelling and edema of the oral cavity, tube was retained post-operatively and extubated subsequently in the intensive care unit.

**In conclusion**, the submandibular method is a novel, alternative method for tracheal intubation in patients with craniomaxillo-facial injuries coming for surgery. It is a low morbidity technique, avoids the complication that occur with tracheostomy and nasal intubation. The procedure has minimal complications and patient's airway is not compromised.
